# The state of smokeless tobacco cessation in a context lacking cessation services: Evidence from Ethiopia

**DOI:** 10.18332/tid/112668

**Published:** 2019-10-29

**Authors:** Mamusha A. Hussen, Edao S. Etu

**Affiliations:** 1 Department of Health, Behaviour and Society, Institute of Health, Jimma University, Jimma, Ethiopia; 2 Department of Public Health, School of Health Science, Goba Referral Hospital, Madda Walabu University, Goba, Ethiopia

**Keywords:** smokeless tobacco, cessation, Borena zone

## Abstract

**INTRODUCTION:**

Cessation attempts for smokeless tobacco (SLT) have been studied in the countries that provide comprehensive cessation services, but there is no evidence about SLT cessation in Ethiopia, where there are no comprehensive tobacco cessation services. The objective of this study was to determine cessation attempts and related factors among daily SLT users.

**METHODS:**

We analyzed the data obtained from a cross-sectional survey of SLT users in Borena zone, Ethiopia, focusing on a subset of 600 daily SLT users. Participants were adult SLT users aged ≥18 years. The dependent variable was SLT cessation attempt. Multivariable logistic regression was performed to identify association between cessation attempts and explanatory variables. Analyses were performed using SPSS version 20.

**RESULTS:**

Overall, 18.5% reported having tried to quit SLT in the past 12 months. In multivariable analyses, SLT cessation attempts were significantly associated with being male (AOR=1.96, 95% CI: 1.13–3.40), current dual-product user (AOR=2.11, 95% CI: 1.31–3.38), being advised by α health professional (AOR=1.82, 95% CI: 1.13–2.92), current knowledge (AOR=1.20, 95% CI: 1.00–1.44), and risk perception (AOR=1.06, 95% CI: 1.02–1.10).

**CONCLUSIONS:**

A low cessation attempt rate among daily SLT users calls for comprehensive cessation intervention. More attention to factors such as knowledge of the health consequences of SLT use, risk perception and health workers advice will be required to encourage cessation attempts.

## INTRODUCTION

There has been substantial progress in reducing tobacco use globally, however, progress has not been uniform across all countries. Low- and middle-income countries in Africa and in other regions continue to have higher death rates attributable to tobacco use^1^. A review on the status of tobacco production and trade in Africa has shown that the area under tobacco cultivation and the importation of tobacco leaf have increased compared to the other parts of the world^2^.

Smokeless tobacco (SLT) products are a non-combustible form of tobacco consumed widely in the continent^3^. The absence of strong tobacco prevention and control policies, coupled with current efforts to expand the SLT market in developing regions, is expected to increase consumption in Africa^4,5^. Given the detrimental effects of secondhand smoke, it is not surprising that SLT has been promoted as an alternative to reduce harm, illness and death caused by smoking tobacco^6-8^. However, SLT product use has been linked to a number of adverse health outcomes such as oral, pharynx and oesophagus cancers^3,9,10^. In Africa, 6485 deaths and substantial disability-adjusted life years (DALYs) lost from oral, pharynx and oesophagus cancers and ischemic heart disease were attributed to SLT use in 2010^3^. This underscores the need to promote SLT cessation. Unless timely action is taken, it is unlikely that the world will reach the World Health Organization (WHO) Member States’ 30% global tobacco use reduction target by 2025.

The WHO Framework Convention on Tobacco Control (FCTC) offers a set of affordable, evidence-based tobacco prevention and control measures. Promoting cessation of tobacco use and providing adequate treatment for tobacco dependence are among the demand-reduction measures recommended by the FCTC^11^. The minimum policy recommendations for a cessation intervention include: cessation advice in primary health care systems, access to free telephone cessation quitlines, and free or low-cost pharmacological therapy. Literature from high-income countries suggests that cessation interventions have been part of successful cessation and are cost-effective^12-16^.

Nevertheless, most African countries, including Ethiopia, have not implemented comprehensive tobacco control policies or practices as suggested by WHO FCTC^4,17,18^. Thus, tobacco use is expected to remain unchanged^1^. In 2014, Ethiopia became a Party to the WHO FCTC and its National Assembly enacted a National Tobacco Control Law^19^. However, there is no comprehensive tobacco cessation intervention for those who seek to quit; the health care system is not oriented in a way to develop and implement tobacco cessation programs^20,21^.

In recent studies conducted in Ethiopia, it was found that 45.3% of adults were current SLT users^22^, and 35.5% intended to quit SLT^23^, whereas only less than one-third of the 45.3% had reported a cessation attempt^22^. Studies on cessation attempts, conducted in areas where there are already comprehensive cessation interventions, have shown that many SLT users attempt to quit and are willing to quit. Factors such as sociodemographic characteristics^24^, health professional advice^24-26^, knowledge of adverse health consequences^26-28^, and tobacco risk perception^29^ were associated with cessation attempts.

In the current study, we aim to identify the level of cessation and associated predictors among SLT users who live in areas where there is no cessation intervention. This research builds on a recent study, which demonstrated that cessation intervention users differed from non-cessation intervention users in their cessation attempts^24^.

Finally, most existing studies do not consider the status of SLT use. They use an aggregate of daily and occasional users instead of examining predictors for cessation attempts, for each group independently^24,27,28,30^. Studies have shown that there is a difference between daily and occasional tobacco users with regard to motivation^31^, perception^32^, and affect^33^. The objective of this study was to determine cessation attempts and related factors among daily SLT users.

## METHODS

### Study settings

This study used data from the second-phase survey of prevalence and factors that influence SLT use among adults in pastoralist communities in the Borena Zone, Ethiopia^22^. The study was conducted in three districts (Yabello, Arero, and Moyale) in the Borena zone, south Ethiopia. Moyale district is situated along Ethiopia’s borders with Somalia and Northern Kenya.

### Design and data source

The data used in this study were derived from a cross-sectional survey conducted 1–20 January 2016, among adult (aged ≥18 years) SLT users in the Borena zone, Ethiopia. The data were collected from 810 randomly selected SLT users. Our analysis focused on 600 daily users^23^.

### Study population and sampling

A multi-stage sampling procedure was used to identify the sample (current SLT users). In the first stage, three districts were selected randomly from the districts in the zone. In the second stage, 30% of the kebeles (the smallest administrative units) were selected from each district. The sample size was allocated proportionally based on the number of SLT users identified by census conducted in the selected kebeles. Finally, the study participants were selected through simple random sampling^23^.

### Data collection

The data were collected using a structured and pre-tested questionnaire. The questionnaire developed in English was translated into the local language (Afan Oromo). A person blind to the English version performed a back-translation to English to check for consistency. Trained researchers collected the data. To maintain data quality, the interviewers were trained, the questionnaire was pilot-tested before the actual data collection, and frequent supervision was conducted during the data collection process^23^.

Ethics approval for this study was not required since the data used were secondary. Ethical clearance for a primary survey was obtained from the research ethics committee of Jimma University (Ref: RPGC/200/2015).

### Measures

#### Outcome variable

The outcome variable was SLT cessation attempts (defined as making any attempt to quit SLT in the past twelve months). Cessation attempts were counted by asking the respondent whether a quit attempt was made during the 12 months prior to the study. The duration of the most recent cessation was categorized as ≤ one month and > one month^24^.

#### Explanatory variables

We included a number of demographic factors: age group (18–30, 31–40, 41–50, and ≥51 years), sex, educational status (unable to read/write, able to read/write, and formal education), occupation (pastoralist, agro-pastoralist, and other), marital status (single, married, divorced, and widowed) and religion (Wakefata, Islam, and Christianity).

Knowledge of health risks associated with SLT use (oral cancer, heart diseases, tooth decay, gum diseases, stomach cancers, and pharyngeal/esophageal cancer) was assessed by six items (Kuder-Richardson Formula 20, KR-20 = 50, on a scale 0–100). The responses to items were summed to create a knowledge scale.

Health risk perception was measured by nine items (Cronbach’s α=0.84) measured on a 5-point Likert scale (e.g. my chances of getting oral cancer by using SLT are rare: 1 = strongly disagree; 5 = strongly agree). All nine items were combined into a sum score to create an overall scale for risk perception.

A modified version of Severson SLT Dependence Scale (SSTDS) was used to assess nicotine dependence. The scale consists of nine items (Cronbach’s α=0.81). The total score for the SSTDS calculated as the sum of all items except the first item. The score ranged from 0 to 18. Higher scores indicate greater dependence. Unlike other scales, SSTDS was developed to address multiple dimensions of tobacco dependence and predicts both craving and withdrawal^34^.

Descriptive social norms were assessed by four items (Cronbach’s α=0.61) rated on a 5-point Likert scale (e.g. most people in my neighborhood chew or snuff tobacco. 1 = strongly agree; 2 = disagree; 3 = undecided; 4 = agree; 5 = strongly agree). The scale score was created by summing all items with the higher score reflecting higher descriptive norm.

### Analysis

We performed descriptive statistics to summarize the participants’ characteristics, SLT related practices, knowledge of health risks, risk perceptions, and norm. We conducted univariable and multivariable logistic regression analyses for the association between cessation attempt and independent variables. Odds ratios (OR) and 95 % confidence intervals (CIs) were calculated for all variables. Variables with p<0.25 in the univariable analysis were included in a multivariable logistic regression model. A p<0.05 was considered statistically significant. All statistical analyses were performed with SPSS version 20.0.

## RESULTS

### Sociodemographic characteristics of respondents

Of the study participants, 67.2% were male, 78.8% were married, and 75% were of the Wakefata religion. Nearly 40% of the respondents were in the age group 31–40 years. The proportion of males (22.3%) who reported cessation attempts was double that (10.7%) of females (Table 1).

**Table 1:** Sociodemographic characteristics of respondents, by cessation status, Borena zone, Ethiopia, 2016 (N=600)

*Variables*	*Respondents*	*Cessation attempt*	
	*n (%)*	*Yes (%)*	*No (%)*
**Age group** (years)			
18–30	25 (4.2)	16.0	84.0
31–40	237 (39.5)	19.0	81.0
41–50	222 (37.0)	20.3	79.7
51–60	116 (19.3)	14.7	85.3
Sex			
Male	403 (67.2)	22.3	77.7
Female	197 (32.8)	10.7	89.3
**Marital status**			
Single	36 (6.0)	19.4	80.6
Married	437 (78.8)	18.0	82.0
Divorced	40 (6.7)	25.0	75.0
Widowed	51 (8.5)	17.6	82.4
**Religion**			
Wakefata	452 (75.3)	18.6	81.4
Islam	116 (19.3)	20.7	79.3
Christian	32 (5.3)	9.4	90.6
**Educational status**			
Unable to read/write	431 (71.8)	16.9	83.1
Able to read/write	109 (18.2)	22.9	77.1
Formal education	60 (10.0)	21.7	78.3
**Occupation**			
Pastoralist	438 (73.0)	21.7	78.3
Agro-pastoralist	123 (20.5)	8.9	91.1
Other*	39 (6.5)	12.8	87.2

* Student, daily laborer, merchant.

### SLT use

All respondents were current daily SLT users. One-fourth of respondents were dual-users of cigarettes and SLT (24.8%). Of all respondents, 18.5% had attempted to quit in the past 12 months. Of these, 52.3% abstained from using tobacco for a period of one month or more during their recent cessation attempt. Among participants who attempted to quit SLT use, 20.3% were in the age group 41–50 years and 22.3% were male (Table 1).

Only 4.8% (29/600) of respondents agreed that SLT use causes oral cancer. The majority of respondents knew that SLT can cause tooth decay (70.5%), mouth sores (43.8%) and gum disease (27.7%). Nevertheless, few respondents identified that SLT use is associated with heart disease (16.8%), stomach cancer (3.2%) and pharyngeal or esophageal cancer (1.5%).

### Perception of risk associated with SLT use, dependence and descriptive norm

The scores of risk perception ranged 11–44 with a mean of 30.26 and standard deviation (SD) of ±6.0. Half of the respondents reported that getting oral cancer is rare, whereas 49.4% indicated that they could get gum disease from SLT use. The score of Severson’s SLT dependence scale ranged from 0 to 18 with a mean 11.1 and ±3.9 SD; the mean score of the social descriptive norm was 11.8 (±3.3 SD).

Table 2 presents participants’ characteristics across dependency score grouped mean rank of tied values. Most of female participants (47.7%) were in the first percentile group. Forty per cent of dual-users and those who had no intention to quit SLT were in the middle percentile and first percentile, respectively. The highest proportion of SLT users in the age group ≥65 years was in the first percentile (high) dependency (48.32%). Compared to dual-users, the proportion of SLT users in the first percentile was higher in the exclusive users (38.1%), although this difference was not significant.

**Table 2:** Participant characteristics according to the mean rank of tied scores for SLT dependency (N=600)

*Variables*	*Percentile SLT dependence score*			*X^2^*	*p*
	*1st Count (%)*	*2nd Count (%)*	*3rd Count (%)*		
**Sex**					
Male	122 (30.3)	142 (35.2)	139 (34.5)	18.370	<0.001
Female	94 (47.7)	58 (29.4)	45 (22.8)		
**Age group** (years)					
18–30	9 (36.0)	11 (44.0)	5 (20.0)	18.431	0.005
31–40	72 (30.4)	96 (40.5)	69 (29.1)		
41–50	79 (35.6)	65 (29.3)	78 (35.1)		
≥**51**	56 (48.3)	28 (24.1)	32 (27.6)		
**SLT use behavior**					
Exclusive user	171 (38.1)	142 (31.6)	136 (30.3)	4.578	0.101
Dual users	43 (28.9)	58 (38.9)	48 (32.2)		
**Duration of recent cessation attempt**					
≤ one month	11 (20.8)	23 (43.4)	19 (35.8)	0.377	0.828
> one month	14 (24.1)	22 (37.9)	22 (37.9)		
**Intention to quit SLT**					
Intend to quit	51 (25.6)	75 (37.7)	73 (36.7)	14.107	0.001
No intention	165 (41.1)	125 (31.2)	111 (27.7)		

Ranks are in ascending order.

### Factor associated with a cessation attempt

In the multiple regression analysis, sex, knowledge of health effects, risk perception, smoking and using SLT (dual-use), and advice from health workers about quitting were positively associated with a cessation attempt. Males were 1.9 times more likely to have made a cessation attempt (AOR=1.96, 95% CI: 1.13–3.40) compared to females. For each unit increase in knowledge on SLT health effects, participants had a 20% higher probability of cessation (AOR=1.206, 95% CI: 1.007–1.445). Similarly, one unit increase in the risk perception leads to a 1.06-fold increase in cessation attempts (AOR=1.065, 95% CI: 1.022–1.109). Dual-users had higher odds of attempting to quit compared with those who use only SLT (AOR=2.11, 95% CI: 1.316–3.383).

In addition, those who received advice from health workers about quitting SLT were 1.8 times more likely to have a cessation attempt (AOR=1.825, 95% CI: 1.138–2.927) (Table 3).

**Table 3:** Multivariable logistic regression model predicting cessation attempt among daily SLT users in Borena Zone, South Ethiopia (N=600)

	*B*	*p*	*NOR*	*95% CI*	
				*Lower*	*Upper*
**Age group** (years)					
18–30	-0.143	0.842	0.867	0.213	3.532
31–40	-0.002	0.995	0.998	0.506	1.968
41–50	0.35	0.305	1.419	0.727	2.770
**Sex**					
Male	0.676	**0.016**	1.966	1.135	3.406
**Occupation of participant**					
Agro-pastoralist	-0.485	0.209	0.616	0.289	1.311
Other	-0.395	0.493	0.673	0.217	2.087
**Educational status**					
Read and write	0.301	0.313	1.351	0.753	2.423
Formal education	0.371	0.313	1.449	0.705	2.980
**Dual-user**					
Yes	0.747	**0.002**	2.110	1.316	3.383
**Advised by health workers to quit**					
Yes	0.602	**0.013**	1.825	1.138	2.927
**SSTDS**	0.067	0.066	1.069	0.996	1.148
**Descriptive social norm**	-0.012	0.726	0.988	0.922	1.058
**Knowledge of health risks**	0.188	**0.042**	1.206	1.007	1.445
**Risk perception**	0.063	**0.003**	1.065	1.022	1.109

Reference groups: age ≥51 years, female, pastoralist, illiterate, do not smoke cigarettes and use SLT simultaneously, received advice from health workers to quit. Maximum SE=0.717, Hosmer-Lemeshaw p=0.201. AOR: adjusted odds ratio. SSTDS: Severson SLT dependence score.

## DISCUSSION

The WHO FCTC recommends promoting cessation of tobacco. Understanding tobacco cessation and related factors help policy-makers design better tobacco control policies and programs. This study assessed predictors of unaided cessation attempts among daily SLT users, which to our knowledge has not been previously quantified in the Ethiopian context. We found that dual use, health professionals’ advice, health effects awareness, and risk perception predicted cessation attempt.

Prevalence of cessation attempt was comparable to the rate reported by a study conducted in India30 but much lower than what was reported in other studies^24,27,28,35-37^. Low rate of cessation attempt is a matter of concern because it can be explained by the absence of comprehensive cessation interventions^21^. As in previous studies^38,39^, cessation attempts are related to predicting successful quitting. This implies that interventions aiming to prevent and control tobacco should encourage cessation attempts and endeavour to search for contributory factors beyond the individual or household level.

Our analysis showed that being male predicted cessation attempts. This is probably due to males being more aware of tobacco-related health risks. In this study, we conducted further analysis of knowledge by sex to explain this finding. It showed that 68.3% of males and 53.8% of females scored above the mean. A previous study also reported that males were more exposed to anti-tobacco messages than females^22^. This finding may indicate that tobacco cessation interventions should give emphasis to women to encourage them to quit.

Our finding is consistent with other studies showing that health professional advice is a key determinant of cessation attempts^24-26^. We found that being advised to quit tobacco by a health professional was associated with increased odds of cessation attempt. This may signify that encouraging health professionals to take advantage of every contact with SLT users, by giving them brief advice, can increase cessation attempts. In areas where other recommended cessation interventions such as pharmacological therapy and cessation advice provided through free telephone helplines are not available, brief advice provided by health professionals is a promising approach to increase cessation rates.

Risk perception is one of the core constructs in health behavior theories^40^; it is an important predictor of health behaviours^41^. In this study, the positive association between risk perception and cessation attempt was in agreement with findings from a previous study conducted among smokers^20^. This finding has implications for tobacco control or cessation interventions in designing health messages. Framing health messages to focus on risk perception could help to increase users risk perception and subsequently cessation attempt.

In addition, we identified that knowledge of negative health consequences of SLT were associated with a previous cessation attempt. Each unit increment in knowledge led to an increase of 20.6% in cessation attempt, and this finding confirmed previous studies results, which revealed that knowledge of negative health consequences is an important predictor of cessation attempt^26-28^. These findings underscore the importance of giving emphasis to increasing knowledge of the health effects of SLT among the public. As reported by different studies, properly framed messages could be successful in increasing cessation success^42^.

### Strengths and limitations

This study reported on SLT users living in a region where there is no organized cessation support, but a need for SLT-use cessation. This is the first study in Ethiopia that addressed unaided SLT cessation attempt and related factors among a large homogenous group. However, it is cross-sectional in design and based on self-reported assessment that might lead to social desirability bias when responding to the questionnaire. Another limitation is that participants’ response about nicotine dependency was self-reported and not validated by serum nicotine or cotinine concentrations.

## CONCLUSIONS

This study demonstrated that in areas where there is no comprehensive cessation intervention, cessation attempts are in need of support. The study highlights cessation should emphasize factors such as knowledge of the negative consequences of SLT, risk perception and health professional advice found to be predictors of cessation attempts. Comprehensive cessation intervention is needed to improve cessation attempts and success, especially in regional settings.
